# Editorial: Enhancing the rehabilitation process with digital technologies - solutions for public health

**DOI:** 10.3389/fpubh.2024.1366077

**Published:** 2024-02-21

**Authors:** Sebastian Rutkowski, Ladislav Batalik, Jannis Papathanasiou, Marco Sacco

**Affiliations:** ^1^Department of Physical Education and Physiotherapy, Opole University of Technology, Opole, Poland; ^2^Department of Physiotherapy and Rehabilitation, Faculty of Medicine, Masaryk University, Brno, South Moravia, Czechia; ^3^Department of Rehabilitation, University Hospital Brno, Brno, Czechia; ^4^Department of Kinesiotherapy, Faculty of Public Health, Medical University Sofia, Sofia, Bulgaria; ^5^Department of Medical Imaging, Allergology, and Physiotherapy, Faculty of Dental Medicine, Medical University of Plovdiv, Plovdiv, Bulgaria; ^6^Institute of Intelligent Industrial Technologies and Systems for Advanced Manufacturing (STIIMA), National Research Council of Italy (CNR), Lecco, Italy

**Keywords:** digital technologies, public health, virtual reality, rehabilitation, telemedicine

In an era where digital technologies are revolutionizing healthcare, the “*Enhancing the rehabilitation process with digital technologies - solutions for public health*” Research Topic provides an essential compendium for exploring innovative solutions. This editorial synthesizes key findings from seven articles, grouped into three thematic categories.

## Application of VR in medicine

The articles within this group highlight the transformative potential of Virtual Reality (VR) in medical interventions. The first study “*eVirtual reality in chemotherapy support for the treatment of physical functions, fear, and quality of life in pediatric cancer patients: a systematic review and meta-analysis*” explores VR's role in alleviating distress and improving the quality of life in pediatric oncology patients (Czech et al.). The systematic review and meta-analysis encompass randomized controlled and crossover studies analyzing the impact of VR on fear, physical functions, and the quality of life in children and adolescents diagnosed with cancer. The results suggest that VR has the potential to become a significant tool in oncological treatment, effectively reducing pain and improving life quality. However, the need for further research to better understand and standardize this therapeutic method was emphasized. Similarly, the article entitled: “*Virtual reality for neurorehabilitation: A bibliometric analysis of knowledge structure and theme trends*” delves into the impact of VR in neurorehabilitation, offering insights into its growing acceptance and application (Guo et al.). It indicates a growing interest and number of publications in this field, primarily from North America and Western Europe. The study identifies key interest areas such as the effectiveness of VR therapy in cognitive and upper limb motor rehabilitation and highlights two main clusters of authors focused on these aspects. It also underscores the need for further research and methodology standardization to fully understand and utilize VR in neurorehabilitation; eventually investigating augmented and mixed reality (AR/MR). The article, “*Breathlessness and exercise with virtual reality system in long-post-coronavirus disease 2019 patients*,” investigates the impact of VR system exercises on breathlessness and functional performance indicators in long-post COVID-19 patients with mild cognitive impairment (Stavrou et al.). The results demonstrated that VR exercises resulted in lower breathlessness scores compared to exercises without VR, suggesting that VR applications are an attractive and safe tool for rehabilitation, potentially enhancing exercise performance and positively affecting patients with both respiratory and cognitive symptoms. Collectively, these studies underscore the need for standardized methodologies and further research to optimize VR's therapeutic potential across various medical disciplines.

## Telemedicine and remote monitoring

Focusing on the expansion of healthcare access, two articles discuss telemedicine and remote monitoring's crucial roles. The article “*A multinational survey of patient utilization of and value conveyed through virtual symptom triage and healthcare referral*” illustrates how virtual triage systems can efficiently navigate patients through healthcare services, enhancing care delivery and optimizing resource allocation (Gellert et al.). The study found that virtual triage effectively redirects users who initially planned to seek inappropriate healthcare levels. It also increases the percentage of patients willing to use telemedicine and engage in virtual health. Users were very satisfied with the virtual triage and experience, with most intending to use it again in the future. Meanwhile, the study “*Inpatient post-COVID-19 rehabilitation program featuring virtual reality—Preliminary results of randomized controlled trial*” (Rutkowski et al.) compares traditional and VR-integrated rehabilitation methods for post-COVID-19 patients, advocating for the broader adoption of novel, technology-driven healthcare solutions. The results indicate significant improvement in both groups regarding exercise performance, measured by the distance in the 6-min walk test. A statistically significant reduction in dyspnoea levels after marching test was also noted, but intergroup comparisons showed no significant differences. Stress level analysis revealed substantial improvements in both groups. Overall, both traditional and innovative VR-based rehabilitation forms demonstrated similar effectiveness in terms of exercise performance and stress levels. These articles collectively signify a shift toward more accessible, efficient, and patient-centered healthcare models.

## Technologies in education

The final thematic category addresses the application of digital platforms in educational contexts. The article “*Remote treatment of developmental dyslexia: how ADHD comorbidity, clinical history and treatment repetition may affect its efficacy*” presents a web-based platform for the remote treatment of developmental dyslexia, illustrating significant benefits regardless of ADHD comorbidity or previous clinical history (Lorusso et al.). The study analyzed the impact of various clinical conditions, including concurrent ADHD, clinical history, and treatment repetition, on therapy effectiveness. Results showed that all children, regardless of comorbid conditions, clinical history, or treatment repetition, achieved significant benefits from the Tachidino platform treatment, effectively eliminating test-retest learning effects. The article “*System Integrated Digital Empowering and teleRehabilitation to promote patient Activation and well-Being in chronic disabilities: A usability and acceptability study*,” examines user experiences with the SIDERA∧B tele-rehabilitation system among patients with chronic heart failure, Parkinson's disease, and chronic obstructive pulmonary disease (Rossetto et al.). Results showed a satisfactory level of technological usability and good ratings in usability subdomains and learnability. Participants rated highly in TAM domains related to “Behavioral Intention,” “Perceived Usefulness,” and “Perceived Ease of Use.” Additionally, the study highlighted that age and disability level were external factors inversely associated with “Perceived Ease of Use.” These studies highlight the versatility and effectiveness of remote therapies and digital tools in managing complex conditions and enhancing patients' autonomy and quality of life.

This Research Topic not only sheds light on the current landscape of digital technologies in rehabilitation and public health but also sets the stage for future innovation and research. From the therapeutic potential of VR to the efficiency of telemedicine and the empowerment provided by educational technologies, the insights from these studies contribute significantly to the understanding and enhancement of rehabilitation processes. As digital technologies continue to evolve, their integration into healthcare promises to advance patient outcomes, access to care, and overall wellbeing, marking a new era in public health and rehabilitation.

## Author contributions

SR: Writing—original draft. LB: Writing—review & editing. JP: Writing—review & editing. MS: Writing—review & editing.

